# Species-specific alterations in *Anopheles* mosquito olfactory responses caused by *Plasmodium* infection

**DOI:** 10.1038/s41598-019-40074-y

**Published:** 2019-03-04

**Authors:** N. M. Stanczyk, V. A. Brugman, V. Austin, F. Sanchez-Roman Teran, S. A. Gezan, M. Emery, T. M. Visser, J. T. Dessens, W. Stevens, R. C. Smallegange, W. Takken, H. Hurd, John Caulfield, M. Birkett, J. Pickett, J. G. Logan

**Affiliations:** 1 Department of Disease Control, Faculty of Infectious and Tropical Diseases, London School of Hygiene and Tropical Medicine, Keppel Street, London, WC1E 7HT UK; 2 Department of Environmental Systems Science, ETH Zürich, Zürich, Switzerland; 3 School of Forest Resources and Conservation, University of Florida, Gainesville, Florida USA; 4 Department of Biointeractions and Crop protection, Rothamsted research, Harpenden, AL5 2JQ UK; 5 Department of Plant Sciences, Laboratory of Entomology, Wageningen University & Research, Wageningen, The Netherlands; 6 Department of Pathogen Molecular Biology, Faculty of Infectious and Tropical Diseases, London School of Hygiene and Tropical Medicine, Keppel Street, London, WC1E 7HT UK; 7 School of Life Sciences, Keele University, Staffordshire, ST5 5BG UK

## Abstract

Mosquitoes infected with malaria parasites have demonstrated altered behaviour that may increase the probability of parasite transmission. Here, we examine the responses of the olfactory system in *Plasmodium falciparum* infected *Anopheles gambiae*, *Plasmodium berghei* infected *Anopheles stephensi*, and *P*. *berghei* infected *An*. *gambiae*. Infected and uninfected mosquitoes showed differential responses to compounds in human odour using electroantennography coupled with gas chromatography (GC-EAG), with 16 peaks triggering responses only in malaria-infected mosquitoes (at oocyst, sporozoite or both stages). A selection of key compounds were examined with EAG, and responses showed differences in the detection thresholds of infected and uninfected mosquitoes to compounds including lactic acid, tetradecanoic acid and benzothiazole, suggesting that the changes in sensitivity may be the reason for differential attraction and biting at the oocyst and sporozoite stages. Importantly, the different cross-species comparisons showed varying sensitivities to compounds, with *P*. *falciparum* infected *An*. *gambiae* differing from *P*. *berghei* infected *An*. *stephensi*, and *P*. *berghei* infected *An*. *gambiae* more similar to the *P*. *berghei* infected *An*. *stephensi*. These differences in sensitivity may reflect long-standing evolutionary relationships between specific *Plasmodium* and *Anopheles* species combinations. This highlights the importance of examining different species interactions in depth to fully understand the impact of malaria infection on mosquito olfactory behaviour.

## Introduction

The malaria parasite, *Plasmodium*, alters behavioural responses of *Anopheles* mosquitoes, however, the degree of effect and the underlying mechanisms are yet to be fully elucidated^1–4^. For decades, the majority of research across multiple *Anopheles*-*Plasmodium* systems has suggested that infected mosquitoes exhibit a different behavioural profile: infected mosquitoes are more likely to bite human or animal hosts, probe more often or repeatedly after disturbance, and show greater behavioural attraction to hosts and nectar (sugar) sources than uninfected mosquitoes^1,5–14^. These behavioural changes are accompanied by a suite of general physiological changes^15–20^. Collective evidence points towards a trend for mosquitoes to show decreased host-seeking and biting activity during the early, non-transmissible oocyst stage of the parasite, and then for these traits to be increased compared to uninfected mosquitoes at the transmissible sporozoite stage^1,5,8,11,13,14^, although two studies did not support this, finding no changes^21,22^. Reduced host-seeking at the non-transmissible stage would reduce the risk of mosquito mortality prior to the possibility of transmission, while increased host-seeking activity at the transmissible stage would result in a greater opportunity for transmission. This would potentially result in changes in transmission dynamics dependant on how widespread this phenomenon is in wild populations^4,23^. A complementary pattern of manipulation of host volatile emissions by *Plasmodium* has been shown to occur in studies with *Plasmodium chabaudi* in a rodent model system^24^ and with *Plasmodium falciparum* in human hosts in the laboratory^25,26^ and field^27–30^, with corresponding increased mosquito attraction to infected hosts^24,27,30,31^.

The mechanisms behind the changes in behaviour of infected mosquitoes remain under investigation, with studies in *Anopheles stephensi* revealing that the same behavioural changes can be instigated through general immune compromise with the bacterium *Escherichia coli*^1^ and suggesting the behaviour may be related to insulin signalling changes taking place upon blood ingestion^32^. It is not yet known what other mechanisms may be involved, or how conserved these may be between different mosquito-*Plasmodium* systems.

Separate from the pathway, an understanding of the phenotypic effects on mosquito behaviour is also highly relevant for vector control. A laboratory study by Smallegange *et al*. showed, for the first time in the important malaria vector, *Anopheles gambiae*, that mosquitoes infected with the transmissible, sporozoite stage of *P*. *falciparum* displayed increased behavioural attraction to human volatiles^8^, and a study by Cator *et al*. demonstrated increased attraction to human odours in a windtunnel by *An*. *stephensi* infected with *Plasmodium yoelii*^1^. These behavioural data suggest that increased behavioural responses to host odour could be mediated through changes in the olfactory system, a hypothesis supported by the latter study’s results that the maxilliary palps exhibited infection-stage dependent differential responses to 1-octen-3-ol, butanoic acid and lactic acid^1^.

In this study, we aimed to further investigate the impact of malaria infection on the responses of infected *Anopheles* mosquitoes. We examined the antennal responses of *An*. *gambiae* and *An*. *stephensi* infected with *P*. *falciparum* and *Plasmodium berghei* to human volatile compounds, with the aim of identifying specific chemicals correlated with altered responses due to infection status. Cross infections of *An*. *gambiae* with *P*. *berghei*, which it does not naturally transmit, were also conducted to determine whether there is mosquito-parasite species-specificity.

## Results

### Anopheles gambiae infected with Plasmodium falciparum

#### EAG

**Figure 1:**
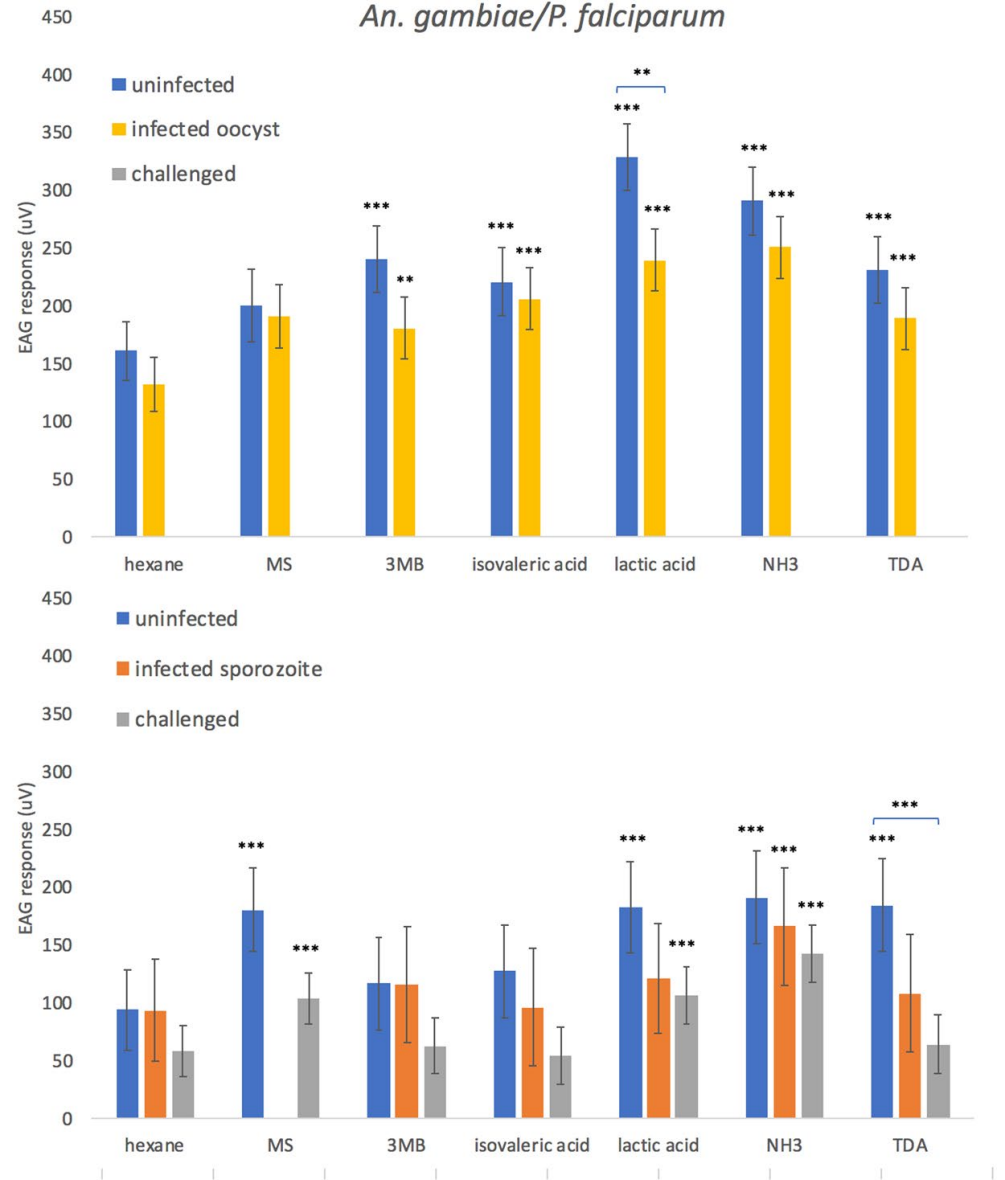
EAG responses (µV) of female *Anopheles gambiae* mosquitoes presented with an uninfected bloodmeal (uninfected) or *Plasmodium falciparum* infected bloodmeal which showed the presence (infected) or absence (challenged) of parasites in oocyst and sporozoite stage mosquitoes. EAG was carried out at (top) 8–10 days post challenge (oocyst stage,) or (bottom) 15–16 days post challenge (sporozoite stage). Treatments were: hexane (negative control), methyl salicylate (MS), 3-methyl-1-butanol (3MB), isovaleric acid, lactic acid, ammonia (NH3) and tetradecanoic acid (TDA). Here, compounds are tagged when significantly different from the negative control (hexane) (***p < 0.001, **p < 0.01, *p < 0.05), or, when indicated with a comparative line, as different between infection statuses. ±Standard Error bars are shown.

Electroantennography (EAG) is a technique by which it is possible to measure antennal receptor responses to volatile compounds. A panel of compounds known to elicit responses in host-seeking *Anopheles* mosquitoes^33–35^, including 3-methyl-1-butanol, isovaleric acid, lactic acid, ammonia, and tetradecanoic acid, were examined individually using EAG in *An*. *gambiae* infected with *P*. *falciparum*. Mosquitoes at the oocyst stage of infection (infection status was ascertained by dissection) showed a significantly lower response to lactic acid than age-matched controls (p = 0.025) (Fig. 1). Challenged mosquitoes (had fed on an infected blood meal but did not develop parasites) exhibited a significantly lower response to tetradecanoic acid at days 15–16 than the age-matched controls (p = 0.012).

Generally, mosquitoes at the sporozoite stage of infection showed a lower sensitivity to the tested volatile compounds than mosquitoes at the oocyst stage of infection, with only ammonia eliciting a response significantly different from the hexane control (Fig. 1), compared to five compounds at the oocyst stage. Uninfected mosquitoes tested on days 8–10 also responded to a greater number of compounds (five) than uninfected mosquitoes tested on days 15–16 (four), showing general differences in sensitivity due to age.

#### GC-EAG

**Figure 2:**
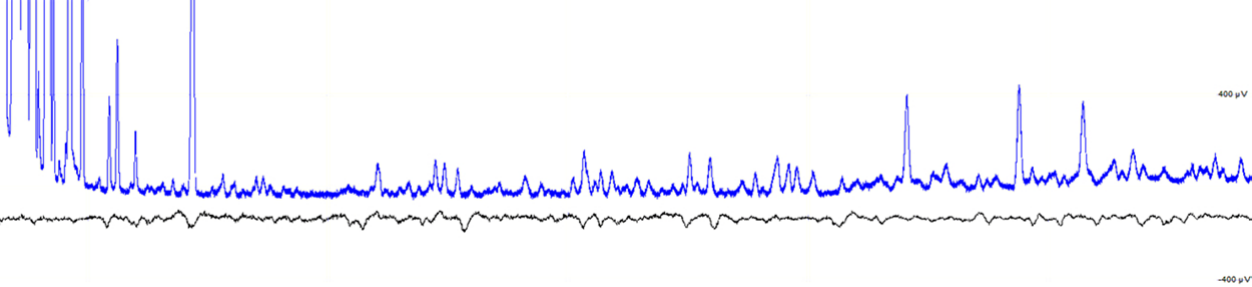
Example GC-EAG trace of an *An*. *gambiae* mosquito infected with the oocyst stage of *P*. *falciparum*, showing GC detection of compounds on the top and mosquito antennal response on the bottom. Where alignment of the top and bottom peaks is consistent for at least 50% of replicates, it is considered an EAG-active mosquito response.

Coupled gas-chromatography-electoantennography (GC-EAG) is a technique used to detect mosquito responses to individual compounds in an odour sample. A concentrated human volatile extract, obtained using headspace collection of the feet of 17 volunteers, was simultaneously detected by flame ionisation detection (GC-FID) and passed over the antennae of individual mosquitoes to record the mosquito olfactory response (see Fig. 2 for example trace). The volatile extract was tested on female *An*. *gambiae* mosquitoes that were at the oocyst stage of infection with *P*. *falciparum* (days 8–10), at the sporozoite stage of infection (days 15–16) and on uninfected mosquitoes of the same ages (age-matched controls). Overall, 37 peaks in human foot odour were detected as EAG active for uninfected and infected mosquitoes at days 8–10 and 15–16 (Table S1) using GC-EAG. Twenty-one of these compounds were active for uninfected or both uninfected and infected mosquitoes (Table S1), however, 16 peaks were specific to infected mosquitoes. Of these, six peaks were specific to oocyst infected mosquitoes, five to sporozoite infected mosquitoes, and a further five peaks triggered responses in both oocyst and sporozoite stage mosquitoes but not the age-matched control group (Table 1). Within these 16 EAG-active peaks, 22 compounds from various chemical groups including the ketones, alkenes, aldehydes and fatty acids were identified by GC-MS (gas chromatography-mass spectrometry).

**Table 1:** Confirmed compounds in human foot odour sample that only female *An*. *gambiae* infected with *P*. *falciparum* responded to by GC-EAG, with mosquitoes responding at the oocyst (O) or sporozoite (S) stage only, or responding at both stages (O&S).

O		S		O&S	
Peak	Compound	Peak	Compound	Peak	Compound
13	Styrene	4	Hexanal	2	Toluene
20	Acetophenone	11	1,3-Dimethylbenzene	6	Unidentified
25	Benzothiazole	30	Verdyl acetate	7	4-Hydroxy-4-methyl-2-pentanone
27	3,4-Dimethylacetophenone		1,4-Diacetylbenzene	16	Phenol
	4-Ethylacetophenone		1,3-Diacetylbenzene		3-Ethyltoluene
28	4-Ethylbenzoic acid	34	Dodecanoic acid		4-Ethyltoluene
36	1-Tetradecanol		DEET	21	Nonanal
		35	1-Hexadecene		
			Tetradecanal		

Where a peak consisted of multiple compounds which could not be separated, all are listed. N = 4–6 replicates for each infection status.

### Anopheles stephensi infected with Plasmodium berghei

**Figure 3:**
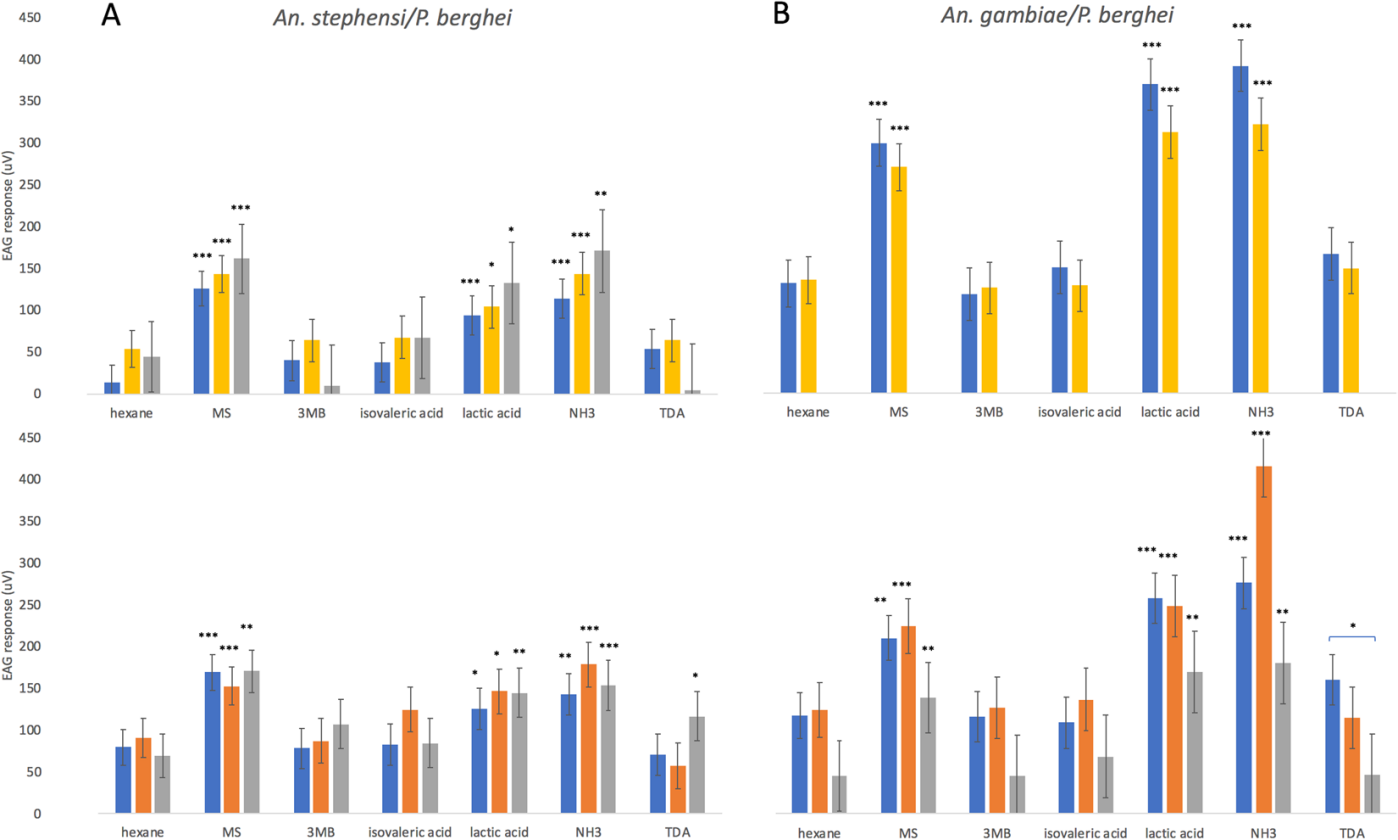
EAG responses (µV) of female *Anopheles stephensi* (**A**) or *Anopheles gambiae* (**B**) mosquitoes presented with an uninfected bloodmeal (uninfected) or *Plasmodium berghei* infected bloodmeal which showed the presence (infected) or absence (challenged) of parasites in oocyst and sporozoite stage mosquitoes. EAG was carried out at (top) 8–10 days post challenge (oocyst stage,) or (bottom) 15–16 days post challenge (sporozoite stage). Treatments were: hexane (negative control), methyl salicylate (MS), 3-methylbutanol (3MB), isovaleric acid, lactic acid, ammonia (NH3) and tetradecanoic acid (TDA). Here, compounds are tagged when significantly different from the negative control (hexane) (***p < 0.001, **p < 0.01, *p < 0.05), or, when indicated with a comparative line, as different between infection statuses. ± Standard Error bars are shown.

To determine whether changes were consistent in a different vector-parasite system, we investigated the EAG response of *P*. *berghei* infected *An*. *stephensi*. There were no significant differences in the level of response between infection status to 3-methyl-1-butanol, isovaleric acid, lactic acid, ammonia, and tetradecanoic acid. However, only challenged *An*. *stephensi* at days 15–16 responded to tetradecanoic acid (Fig. 3A) compared to a solvent control.

A further compound selected from the literature, benzothiazole^24,36^, was additionally tested by EAG dose response after it also appeared as an important compound in GC-EAG experiments above (Fig. S1). Infected *An*. *stephensi* at the oocyst stage showed significantly greater responses to the highest concentration of benzothiazole (1%) than age-matched controls (p = 0.0264), and these also showed a significantly greater response to this concentration than mosquitoes at the sporozoite stage of infection (p = 0.045) (Fig. S1).

### Anopheles gambiae infected with Plasmodium berghei

To determine whether there was a species-specific alteration in olfactory responses, we performed a cross infection of *An*. *gambiae* with *P*. *berghei*. We found no significant differences in EAG responses between infected and uninfected mosquitoes to any of the compounds, and overall responses to compounds compared to the control were not different between statuses (Fig. 3B). However, challenged mosquitoes at days 15–16 showed significantly lower responses to tetradecanoic acid (p = 0.0427) than age-matched controls.

## Discussion

Here we have demonstrated that uninfected and *P*. *falciparum*-infected *An*. *gambiae* mosquitoes respond differently to the compounds within natural human volatile extracts when analysed by GC-EAG, with a third of the total number of compounds triggering responses only in infected mosquitoes: these included compounds also known to be emitted differentially by *Plasmodium*-infected hosts, such as benzothiazole, hexanal, toluene and nonanal^24,26,29,30^. This result suggests that infection with *P*. *falciparum* alters the olfactory sensitivity of mosquitoes to specific compounds including those known to elicit behavioural responses, such as benzothiazole and nonanal^24,37^. Altered sensitivity of detection could explain the increased attraction to hosts at the sporozoite stage and decreased attraction at the oocyst stage of the *Plasmodium* life cycle found in previous studies^1,5,8,11,13^.

When examining individual compounds in *P*. *falciparum* infected *An*. *gambiae*, we found direct differences in EAG responses to lactic acid between mosquitoes at the oocyst stage of infection and uninfected mosquitoes, with infected mosquitoes showing lower responses (Fig. 1). As lactic acid is a well-known mosquito attractant^38^, a lower response during oocyst stage infection correlates with the hypothesis that non-transmissible parasites might benefit from reduced host-seeking and the associated risk of mortality^2^. There also appeared to be an effect of immune challenge upon response to tetradecanoic acid, with ‘challenged’ *An*. *gambiae* (mosquitoes that had taken a *P*. *falciparum* infected bloodmeal without becoming infected) expressing a lower EAG response than age-matched controls at 15–16 days following bloodmeal exposure. This result confirms the effect of immune challenge on mosquito olfaction found in *An*. *stephensi* in a previous study^1^, but suggests that the specific effects on the antennal sensitivities to compounds may alter differentially dependant on both the mosquito and *Plasmodium* species involved.

In order to examine species effects, we also performed EAG experiments on *An*. *stephensi* infected with *P*. *berghei*. In contrast to the results seen with *An*. *gambiae* and *P*. *falciparum*, *An*. *stephensi* with *P*. *berghei* oocyst-stage infection did not show different responses to lactic acid, but showed a greater response than age-matched controls to benzothiazole, a compound known to influence mosquito attraction^36^ that was recently found to be produced in lower quantities by *P*. *chabaudi*-infected rodent hosts, which were more attractive to *An*. *stephensi*^24^. The greater sensitivity towards this compound by oocyst-infected *An*. *stephensi* in our study suggests that infected mosquitoes are capable of detecting specific host-derived volatiles at lower concentrations than uninfected mosquitoes. It is unknown, however, how this sensitivity manifests behaviourally, since a prior study examining *An*. *stephensi* infected with *P*. *berghei* found no alterations in probing duration^39^. Alterations due to immune challenge with an infected blood meal were not seen here in *An*. *stephensi*, despite demonstration of this in a previous study with *P*. *yoelii*^1^.

The overall patterns of significant responses versus a solvent control shifted between infection statuses in certain species. For example, comparing oocyst-stage infections, *P*. *falciparum*-infected *An*. *gambiae* responded to a larger panel of compounds than *P*. *berghei*-infected *An*. *stephensi*, while at the sporozoite stage the former showed a reduction in the range of detected compounds, with lactic acid no longer detected by infected individuals. This shift in which compounds are detected at the sporozoite stage potentially runs counterintuitive to the hypothesis that mosquitoes will bite more if they detect compounds more strongly, and the previously observed increased behaviour at this transmissible stage in *An*. *gambiae*^8^. However, as *An*. *gambiae* infected at the sporozoite stage with *P*. *falciparum* still show detection of ammonia, it is possible that mosquitoes at this stage in the infection cycle rely more heavily on a select few volatile cues – this should be investigated further by focusing on behavioural responses to blends. Furthermore, as the electroantennograms with *An*. *stephensi* do not show this reduction in detection at the sporozoite stage, this is possibly an effect which varies between mosquito species and is dependent on the effect of parasite infection on the host physiology, or alternatively the effect may have been caused by variation in parasite load. Due to the logistical difficulties of artificially infecting *An*. *gambiae* with *P*. *falciparum*, the number of sporozoite infected mosquitoes tested in this study was low, and there was also an overall correlation between increased mosquito age and reduction in response which may have acted as a confounding factor.

Overall, *An*. *gambiae* infected with *P*. *falciparum* and *An*. *stephensi* infected with *P*. *berghei* both show alterations in sensitivity to different compounds, suggesting that the effect of *Plasmodium* infection on mosquitoes is species-specific. This may explain why some studies found no effect on vector behaviour in their *Plasmodium*-mosquito systems^21,22^. If certain combinations of mosquito and *Plasmodium* species do not show altered biting or attraction behaviour, it is likely that the change in olfactory responses seen here in the antennae of *An*. *gambiae* and *An*. *stephensi*, and previously in the maxilliary palps of *An*. *stephensi*^1^, would also not occur in those systems. In addition, there may be variation in mosquito response to infection even within the same system, given that a recent study on *An*. *gambiae and P*. *falciparum* found no differences in behavioural responses^22^. While the contrast in findings to previous behavioural studies, which showed altered responses in *An*. *gambiae* infected with *P*. *falciparum*^8,12,40^, could be due to different methodologies, it is also possible that genetic and environmental factors play a large role in the development of the altered behavioural phenotype. Additionally, mosquitoes and *Plasmodium* from varying geographical locations may show contrasting responses. The cross-infection of *An*. *gambiae* with *P*. *berghei* in our study showed a similar profile of responses to *An*. *stephensi* infected with *P*. *berghei*, but unfortunately parallel results for benzothiazole could not be presented due to a low number of replicates. *Plasmodium falciparum* and *P*. *berghei* have been shown previously to have different effects on the *An*. *gambiae* immune system^41^ and it is uncertain whether the similarities between the two *P*. *berghei* infections are due to the *Plasmodium* species being responsible for the majority of the olfactory alterations, irrespective of mosquito species, or if this may be an artefact due to the lower incubation temperatures used when infecting with *P*. *berghei* in both cases – it is difficult to draw direct conclusions in this case.

This study has provided more evidence indicating that malaria parasite infection can influence the physiology of *Anopheles* mosquitoes, in this case by altering their sensitivity to specific host-derived compounds. The differences observed between the results of each *Plasmodium*-*Anopheles* combination highlight the importance of conducting electrophysiological and by extension behavioural studies using different model systems in both the laboratory and field to fully elucidate the impact of parasite infection. Such system-specific differences are likely to result from a long evolutionary association between certain mosquitoes, parasites and hosts and hint at possible approaches for exploitation of tailored compound blends for use in vector surveillance and control strategies.

## Methods

### Mosquitoes and *Plasmodium*

#### Mosquito rearing

*Anopheles gambiae* s.s. N’guesso strain (originally obtained from Imperial College, London, UK) and *Anopheles stephensi* SDA 500 strain were maintained at the London School of Hygiene and Tropical Medicine (LSHTM, UK). Both species were reared at a relative humidity of 60–70% and a 12:12 light:dark photoperiod and maintained at a temperature of 27 ± 2 °C. Larvae were fed with Tetramin® (Melle, Germany) tropical fish flakes, and adults provided with 10% glucose solution, with weekly feeds of human blood (collected from volunteers at LSHTM with informed consent obtained; LSHTM ethics committee reference number 5520) using a Hemotek© artificial membrane feeding system (Hemotek Ltd, Blackburn, UK). Females for use in experimental infections were allowed to emerge in a separate cage and supplied with 10% glucose only.

#### Mosquito infection with P. falciparum

*Plasmodium falciparum* NF54 parasites were cultured in human serum (pooled, sterile-filtered blood from males of blood group AB) as previously described^42,43^. Asexual blood stage parasites were synchronized by a 5% D-sorbitol (wt/vol) treatment for 10 min at 37 °C. Synchronous ring stage parasites at 5% haematocrit were cultured to 6–10% parasitaemia, at which point sexual development was triggered via starvation-induced stress^43^. Trophozoite cultures were diluted two- to three-fold the following day in T25 flasks at 3–5% haematocrit such that each flask contained 0.5–2% gametocytes in a final volume of 10 ml medium. For infection, freshly drawn human whole blood was washed in RPMI and resuspended in prewarmed serum to give a packed cell volume of 40%. 14–17 day old gametocyte cultures (0.5–2% gametocytaemia, 5% haematocrit) were gently agitated for cell resuspension, transferred to prewarmed tubes and centrifuged to pellet for 5 minutes at 1800 x g at 37 °C. The parasite pellet was then mixed with an equal volume of serum to give a parasite/serum mixture that was subsequently diluted with 3–9 times its volume with fresh whole blood/serum, constituting the final infectious feed. Three to eight-day old nulliparous female mosquitoes were allowed to feed on the infectious feed via a Hemotek© (1 ml reservoirs) for 10–15 minutes. A control group of mosquitoes from the same batch was fed using only the uninfected blood/serum mixture and maintained alongside the infected batch. After blood-feeding, mosquitoes were maintained at 25 °C at ~70% relative humidity with constant access to 10% glucose.

#### Mosquito infection with P. berghei

*Plasmodium berghei* ANKA clone 234 parasites were maintained as cryopreserved stabilates or by serial blood passage in 6–8 week old female CD1 mice and regular mosquito transmission. Mosquitoes were infected with *P*. *berghei* ANKA by feeding directly on parasite-infected mice (anesthetised with Rompun and Ketaset). Briefly, hyper-reticulocytosis was induced 3 days before infection by treating mice with 200 μl intraperitoneal (i.p.) phenylhydrazine chloride (PHz; 6 mg.ml^−1^ in PBS; ProLabo, UK). PHz-treated mice were infected by i.p. injection of parasitized blood (approximately 200 μl of blood at 1% parasitaemia), and infections were monitored by examination of Giemsa-stained tail blood smears. At 3 days post-infection, anaesthetized mice were exposed to pots containing 50–70 starved female anopheline mosquitoes. Blood-fed mosquitoes were maintained on 10% (w/v) fructose, 0.05% (w/v) p-aminobenzoic acid at 20 °C and 60% relative humidity. Control mosquitoes of the same age were fed on uninfected mice and kept under the same conditions.

Ethics. Animal work was conducted under UK Home Office license and approval in accordance with the United Kingdom Animals (Scientific Procedures) Act 1986 implementing European Directive 2010/63 for the protection of animals used for experimental purposes. All methods were carried out in accordance with relevant guidelines and regulations and approval was obtained from the LSHTM Animal Welfare Ethics Review Board. Animal welfare was assessed daily and animals were humanely killed upon reaching experimental or humane endpoints.

#### Confirmation of infection

Infection status was ascertained for all mosquitoes by light microscopy. Once the head was separated from the body, the midgut was removed and examined for the presence of oocysts after staining with mercurochrome. Salivary glands were excised to check for the presence of sporozoites. Mosquitoes were then designated as either infected (oocyst or sporozoite stage) or challenged if there were no visible oocysts or sporozoites but the mosquito had fed on an infected blood meal. Age-controlled uninfected mosquitoes were also dissected for control purposes.

### Volatile collection and electrophysiology

#### EAG

Electroantennography was used to examine the antennal responses of infected and uninfected mosquitoes to known attractive host compounds at day 8–10 (oocyst stage) and 15–16 (sporozoite stage) post bloodmeal in *An*. *gambiae* infected with *P*. *falciparum*, *An*. *stephensi* infected with *P*. *berghei*, and *An*. *gambiae* infected with *P*. *berghei*. This approach measures the depolarisation across the antennal membrane in response to a volatile stimulus. The mosquito was prepared by removing the head after being cold-anaesthetised; the tips of the antennae were then excised using a scalpel, and the proboscis and maxilliary palps removed to prevent movement during experiments. The head was mounted on the point of a tapered glass electrode filled with Ringer’s solution (7.55 g NaCl, 0.64 g KCl, 0.22 g CaCl_2_, 1.73 g MgCl_2_, 0.86 g Na_2_HCO_3_, and 0.61 g Na_3_PO_4_ l^−1^ water), and the tips of the antennae positioned into a glass recording electrode (also containing Ringer’s solution). These glass electrodes were mounted on silver electrodes connected to a probe and an IDAC4, which allowed signals from the antennae to be recorded using EAD 2000 software (Syntech®, Buchenbach, Germany). Compounds were used at concentrations known to elicit EAG responses from our preliminary tests and in fitting with recommendations from the available literature^44^. The test compound (10 µl) was dotted onto a strip of filter paper, with hexane as a negative control and methyl salicylate as a positive control to test for responsiveness of the preparation (Table 2). A replicate consisted of each treatment tested on a single female mosquito (Table 3). All experiments were carried out in daylight, in the first 8 hours post-scotophase.

**Table 2:** List of compounds used in EAG treatments.

Chemical name	Supplier	% Purity	Tested concentrations (%, in hexane where diluted)
3-Methyl-1-butanol	Sigma Aldrich	99	0.00001
Methyl salicylate	Sigma Aldrich	99	0.01
Benzothiazole	Sigma Aldrich	96	1, 0.1, 0.01, 0.001, 0.0001
Tetradecanoic acid	Sigma Aldrich	99	0.00001
Ammonium hydroxide	Sigma Aldrich	28–30	28
Isovaleric acid	Sigma Aldrich	99	0.00001
L-(+)-Lactic acid	Fisher	90	90

**Table 3:** Number of EAG replicates for each status of infection.

Fig.	Mosquito	Parasite challenge	Oocyst control	Oocyst challenged	Oocyst infected	Sporozoite control	Sporozoite challenged	Sporozoite infected
1	*An*. *gambiae*	*P*. *falciparum*	15	—	18	10	21	5
3	*An*. *gambiae*	*P*. *berghei*	14	—	14	14	5	9
3	*An*. *stephensi*	*P*. *berghei*	14	5	12	11	11	8
S1	*An*. *stephensi*	*P*. *berghei*	12	5	11	10	7	7

#### Volatile collection and chemical identification

Human volatiles were collected from the feet of 17 volunteers. Volunteers placed their feet in individual 25 × 38 cm plastic roasting bags (toastabags®, Planit Products Ltd., Malvern, UK), which were sealed to be airtight. A purified airflow was then pumped into the top of the bag at 1.2 l/min, and pulled out of the bottom of the bag through a polymer filter (Porapak Q, mesh size 50/80, Supelco Analytical) at 0.8 l/min for 2 hours. Volatile odours were collected on the polymer filter, then eluted with 750 µl redistilled diethyl ether. Both feet of each volunteer were used, to give a total of 34 samples. Each sample was concentrated to 50 µl under nitrogen, and the samples were pooled. The total volume of 1.7 ml was then concentrated further to 400 µl, in order to provide a concentration representing 10 minutes of volatile collection/µl. This sample was used for all EAG work and was analysed by coupled gas chromatography-mass spectrometry (GC-MS) performed on a Micromass Autospec Ultima magnetic sector mass spectrometer, attached to an Agilent 6890 N GC (non-polar HP1 column 50 m length x 0.32 mm inner diameter x 0.52 µm film thickness, Agilent, Santa Clara, U.S.A.) equipped with a cool-on-column injector. Ionization was by electron impact (70 ev, 220 °C). The GC oven temperature was maintained at 30 °C for 5 min, then programmed at 5 °C/min to 250 °C and held for 21 min). The identity of compounds of interest was confirmed by co-injecting the odour sample and the compound to be tested onto the GC, with the aim of doubling the area of the GC peak detected.

#### GC-EAG

Coupled gas-chromatography-electroantennography (GC-EAG) was performed as described by Logan *et al*.^37^. Briefly, 2 µl of the pooled volunteer foot volatile collection was injected on to an HP1 column (50 m, 0.32 mm ID, 0.52 film thickness) with a cool-on column injector, and split so that half the sample was detected by the GC-FID (Chemstation, Agilent, Santa Clara, U.S.A.) and the other half was passed over the antennae of the mosquito. This formed a combined trace (Fig. 2) where mosquito responses were directly aligned with the chemicals causing them. The mosquito was prepared as above, and for the GC method, the oven started at 30 °C, was held for 1 min, then raised by 5 °C/min to 100 °C, then by 10 °C/min to 230 °C and held for 1 min. 4–6 replicates were carried out for *P*. *falciparum* infected and uninfected *An*. *gambiae* mosquitoes at day 8–10 (oocyst stage) and 15–16 days (sporozoite stage) post-bloodmeal. Conserved responses to FID peaks across replicates were aligned using a lightpad.

### Statistical analysis

Several linear mixed models were analysed for each of the performed experiments. In all cases, the response variable corresponded to microvolts (µV). The data for this experiment were evaluated with a three-way analysis of variance with the main fixed factors of *type*, *status* and *trt* together with all interactions. The factor *type* has the levels of infection type (challenged, infected, control), the factor *status* describes the infection status (oocyst, sporozoite), and the factor *trt* corresponding to different chemicals and their concentration. The model fit also included the random factors of *replicate* and *sequence*, where the replicate represents a run that contained, depending on the experiment, between 6 to 32 treatment combinations (*status-type-trt*), and the order of these applications within a replicate were described by the factor *sequence*. Significance of the different factors and interactions were done with an approximate F-test where the degrees of freedom were estimated using the Kenward-Rogers method. In addition, due to this sequence of treatments, repeated measures were modelled within each replicate by considering a homogeneous autoregressive of order 1 error structure. Comparisons of predicted means of the different levels of the combination factor *status-type-trt* for each of the experiment were evaluated by Least Significant Difference. All models were fitted using proc mixed as implemented in SAS version 9.4 (SAS Institute, Cary, NC, USA). No departures from normality were observed and a significance level of 0.05 was considered for all tests. Significant comparisons were reported for each *status-type* combination where the treatments were significantly different from the control, or if there were differences in response to the same treatment across different statuses or types. Comparisons of treatments across status or type were only made when there was no difference between the means of the relevant negative controls, i.e. for control and infected oocysts responses to the negative controls were not significantly different.

This study and the experimental protocols herein were approved by the LSHTM ethics committee (reference numbers 6435 and 5520) and conducted according to committee guidelines and regulations.

## Supplementary information

Supplementary Information
