# Supplementary material for: Species-specific alterations in Anopheles mosquito olfactory responses caused by Plasmodium infection
Source: Sci Rep. 2019 Mar 4;9:3396. doi: 10.1038/s41598-019-40074-y (PMC6399344; doi:10.1038/s41598-019-40074-y)
Supplement: Supplementary file 1 — Supplementary Information [file 41598_2019_40074_MOESM1_ESM.docx]

**Title:** Species-specific alterations in *Anopheles* mosquito olfactory responses caused by *Plasmodium* infection

**Authors**: Stanczyk NM^1,2^(CO), Brugman VA^1^(CO), Austin V^1^, Sanchez-Roman Teran F^1^, Gezan SA^3^, Emery M^4^, Visser TM^5^, Dessens, JT^6^, Stevens W^1^, Smallegange R^5^, Takken W^5^, Hurd, H^7^, John Caulfield^4^, Birkett M^4^, Pickett J^4^, Logan JG^1^ *

**Affiliations**:

^1^ Department of Disease Control, Faculty of Infectious and Tropical Diseases, London School of Hygiene and Tropical Medicine, Keppel Street, London, WC1E 7HT, UK

^2^ Department of Environmental Systems Science, ETH Zürich, Switzerland

^3^ School of Forest Resources and Conservation, ^­^University of Florida, Gainesville, Florida, USA

^4^ Department of Biointeractions and Crop protection, Rothamsted research, UK

^5^ Department of Plant Sciences, Laboratory of Entomology, Wageningen University & Research, The Netherlands

^6^ Department of Pathogen Molecular Biology, Faculty of Infectious and Tropical Diseases, London School of Hygiene and Tropical Medicine, Keppel Street, London, WC1E 7HT, UK

^7^ School of Life Sciences, Keele University, Staffordshire, ST5 5BG, UK

Supplementary Material

Table S1: GC-EAG peaks in volatile odour samples collected from human feet confirmed by co-injection. *An. gambiae* infected with *P. falciparum* infection stages tested with EAG: O = oocyst, S = sporozoite, U1 = uninfected control for oocyst stage, U2 = uninfected control for sporozoite stage. Where a peak consisted of multiple compounds which could not be separated, all are listed. N = 4-6 replicates for all treatments. KI = Kovats Index.

| **Peak** | **Compound** | **KI** | **Infection stage responding** | **Peak** | **Compound** | **KI** | **Infection stage responding** |
| --- | --- | --- | --- | --- | --- | --- | --- |
| 1 | N,N-Dimethylformamide | 737 | U1+U2+O+S | 20 | Acetophenone | 1041 | O |
| 2 | Toluene | 754 | O+S | 21 | Nonanal | 1085 | O+S |
| 3 | Unidentified | 778 | U2+S | 22 | Undecane | 1099 | U2 |
| 4 | Hexanal | 777 | S |  | 2-Ethylhexanoic acid | 1104 | U2 |
| 5 | Unidentified | 788 | U1+S | 23 | Camphor | 1124 | U2 |
| 6 | Unidentified | 799 | O+S | 24 | 1-Nonanol | 1159 | U1+O |
| 7 | 4-Hydroxy-4-methyl-2-pentanone | 815 | O+S |  | Unidentified | 1173 | U1+O |
| 8 | 2,4-Dimethylheptane | 827 | U2+S | 25 | Benzothiazole | 1202 | O |
| 9 | Unidentified | 832 | U1 | 26 | Unidentified | 1261 | U1+U2 |
| 10 | Ethylbenzene | 852 | U2 | 27 | Unidentified | 1288 | O |
| 11 | 1,3-Dimethylbenzene | 860 | S | 28 | 4-Ethylbenzoic acid | 1335 | O |
| 12 | 4-Methyloctane | 868 | U1+S | 29 | Tetradecane | 1399 | U1 |
| 13 | Styrene | 878 | O | 30 | Verdyl acetate | 1404 | S |
| 14 | Nonane | 902 | U2 | 31 | Geranylacetone | 1434 | U1 |
| 15 | Benzaldehyde | 934 | U1 | 32 | 1-Dodecanol | 1460 | U1 |
| 16 | Phenol | *956* | O+S | 33 | Pentadecane | 1500 | U1 |
|  | 3-Ethyltoluene | *955* | O+S | 34 | Dodecanoic acid | 1535 | S |
|  | 4-Ethyltoluene | *957* | O+S | 35 | Unidentified | 1590 | S |
| 17 | 2-Ethyltoluene | 969 | U1+S | 36 | Unidentified | 1662 | O |
| 18 | Decane | 998 | U2+S | 37 | Isopropyl myristate | 1810 | U2 |
| 19 | Limonene | 1026 | U1+O+S |  |  |  |  |


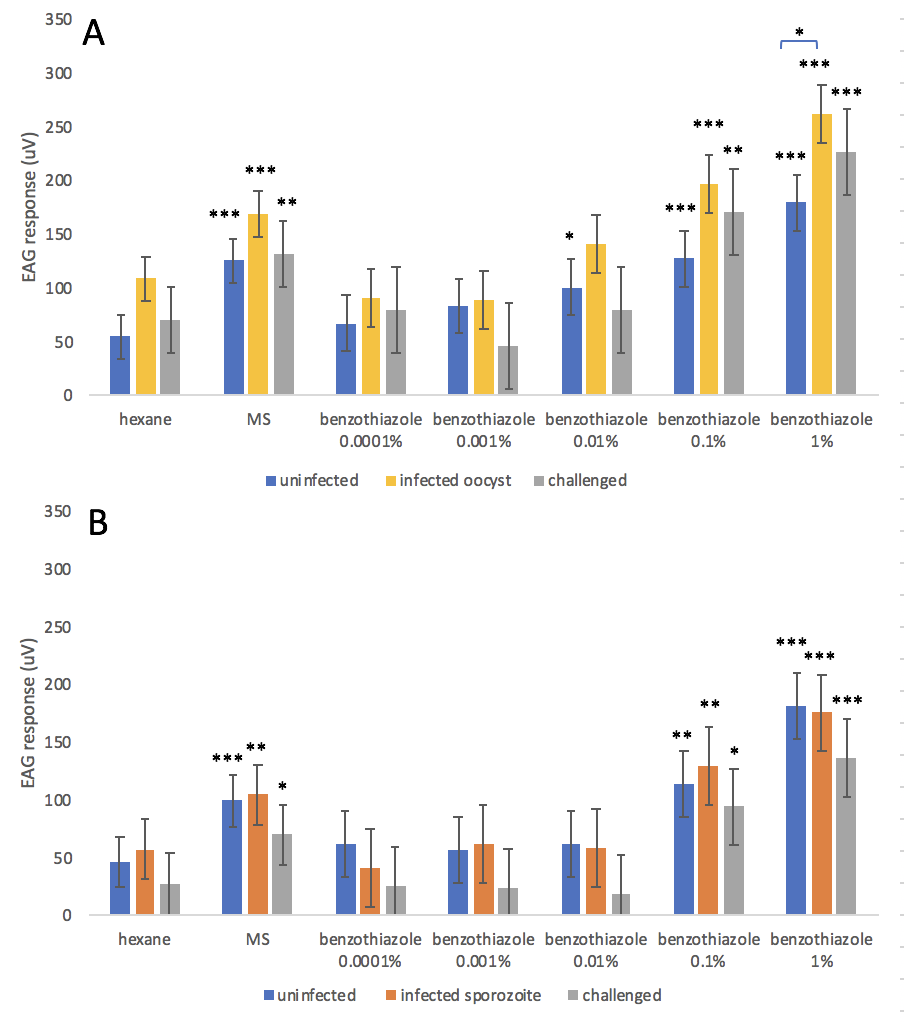


Fig S1. Dose response EAG to benzothiazole of female *An. stephensi* mosquitoes given an uninfected bloodmeal (control) or *P. berghei* infected bloodmeal which showed the presence (infected) or absence (challenged) of parasites upon dissection. EAG was carried out at: A) 8-10 days post challenge (oocyst stage), or B) 15-16 days post challenge (sporozoite stage). Here, compounds are tagged when significantly different from the negative control (*** p<0.001, ** p<0.01, * p<0.05) or, as indicated with a comparative line, when different between infection status.
